# Connecting the conceptual dots in embodied cognition: A commentary on “How body balance influences political party evaluations: a Wii balance board study”

**DOI:** 10.3389/fpsyg.2015.00853

**Published:** 2015-06-30

**Authors:** Andrew D. Wilson, Sabrina Golonka

**Affiliations:** School of Social, Psychological and Communication Sciences, Leeds Beckett UniversityLeeds, UK

**Keywords:** embodied cognition, psychological mechanisms, conceptual metaphors, conceptual grounding, task analysis

Embodied cognition is the hypothesis that things other than the brain contribute meaningfully to the structure of our cognition. A critical (and often ignored) corollary of this is that any example of embodied cognition in action needs an account of exactly how these distributed elements are connected to one another so that they might affect each other. A given embodied cognition effect therefore needs to come with a plausible path along which information can flow (Wilson and Golonka, 2013). Identifying this path is difficult, and it's not always possible to do so immediately; this is not a problem, and simply indicates a place for future research. However, when the only viable pathway is tested and fails, there *is* a problem and it suggests that there is something fundamentally wrong with the analysis of the task.

Dijkstra et al. (2012) asked people to attribute neutral political statements to Dutch political parties spanning the left/right political spectrum. Participants' posture was surreptitiously altered to be slightly (2%) left or right of center. The hypothesis was that leaning to the left/right would prime the conceptual metaphor “left wing/right wing.” This would then make the primed metaphor more likely to be used to interpret the otherwise neutral political statements, making people more likely to attribute the statement to a party they know to be left/right wing. This hypothesis would be supported if leaning left/right made people more likely to select a party they believed was left wing/right wing.

When leaning left, people were indeed more likely to attribute the statements to left-wing political parties (*p* < 0.01), but when leaning right there was only a trend to attribute the statements to right wing parties (*p* = 0.07). The authors concluded that the results support the hypothesis.

There is a problem, however. The effect was only apparent when the political party's affiliations were coded according to their *actual* affiliation (Dijkstra et al., Figure 2). The effect vanishes entirely when the party affiliations were coded according to participants' *knowledge* of that affiliation. This is because the participants were only able to correctly identify the affiliation of fewer than half of the 10 parties, and, in general, scored very low on measures of interest and knowledge of politics. The participants were missing a critical part of the puzzle (knowing which parties were left or right wing)—so how were they able to connect the conceptual dots to generate the observed effect?

The authors suggest that the problem was that the 2D grid task which measured knowledge (left wing/right wing by progressive/conservative) was too hard (see also Dijkstra et al., 2014). As evidence they show a small but significant improvement in affiliation identification in a second group of participants using a 1D left/right classification (5.97/10, vs. 4.57/10 for the first group). However, the first group never completed the easier classification task and the second group never matched statements to parties. There is also the fact that the first group showed very low interest and knowledge in politics in general, suggesting they may not have benefited from the different task. At the very least, the onus is on the authors to replicate their effect with the alternative measure of knowledge, and show that the key interaction now shows up when the data are coded according to the results of that measure. We are currently left to infer that this would happen.

Dijkstra et al., did the right thing in measuring the critical link (knowledge of political affiliation) but they have failed to identify that the overall pattern of results as they stand do not and cannot support their conceptual embodiment hypothesis. Their results show there is no plausible mechanism to explain the effect, no path along which information could flow. Whatever happened, it was not caused by a metaphor being grounded in postural sway.

## Analysis

We think a problem with this line of research lies in the absence of a detailed task analysis (Wilson and Golonka, 2013). What, precisely, is the task facing the observer, and what are the critical resources available to solve the task at hand? These questions are never addressed in this or any related embodiment research. For example, why is posture implicated in the task at all? The left wing/right wing convention comes from the spatial organization of the French legislature. Why would this be grounded in postural sway? And why would a small change in posture (on the order of a perfectly ordinary, moment-to-moment postural adjustment) affect political attribution? There may be a story, but it is not yet forthcoming.

Contrast this to some of the most successful embodied cognition programmes around. The embodied solutions to things such as the A-not-B error (Thelen et al., 2001) and the outfielder problem (McBeath et al., 1995) are grounded in a meticulous analysis of what is absolutely required to solve a given task, as well as an account of how these resources are assembled to implement that solution. The net result is a model which accounts for large amounts of pre-existing data and which makes testable (and successful) predictions of large, robust effects. You cannot make the A-not-B error without reaching like an infant, and so reaching is a critical part of the overall explanation. You can, however, attribute neutral political statements to parties without leaning in any direction. Posture is therefore not a task-critical element and there is, as yet, no adequate account in the literature as to why our conceptualization of the political world should reflect it.

Embodied cognition is the hypothesis that the form of our behavior emerges from a distributed set of task resources working together to solve a task. Embodied cognition researchers therefore have an obligation to identify both the composition and the organization of this distributed system, in order to account for how there might possibly be more to us than our brains. Without this ground work, our explanations for our data are massively under-constrained and we might fail to notice that the dots could not possibly have been connected the way we claimed.

### Conflict of interest statement

The authors declare that the research was conducted in the absence of any commercial or financial relationships that could be construed as a potential conflict of interest.
